# Transcription Factor-Based Biosensors for Detecting Pathogens

**DOI:** 10.3390/bios12070470

**Published:** 2022-06-29

**Authors:** Yangwon Jeon, Yejin Lee, Keugtae Kim, Geupil Jang, Youngdae Yoon

**Affiliations:** 1 Department of Environmental Health Science, Konkuk University, Seoul 05029, Korea; ywjun69@gmail.com (Y.J.); cat770550@naver.com (Y.L.); 2 Department of Environmental & Energy Engineering, University of Suwon, Hwaseong 18323, Korea; kkt38@suwon.ac.kr; 3 School of Biological Sciences and Technology, Chonnam National University, Gwangju 61186, Korea

**Keywords:** biosensors, pathogens, biodetection, TF-based biosensors, cell-based biosensors, cell-free biosensors

## Abstract

Microorganisms are omnipresent and inseparable from our life. Many of them are beneficial to humans, while some are not. Importantly, foods and beverages are susceptible to microbial contamination, with their toxins causing illnesses and even death in some cases. Therefore, monitoring and detecting harmful microorganisms are critical to ensuring human health and safety. For several decades, many methods have been developed to detect and monitor microorganisms and their toxicants. Conventionally, nucleic acid analysis and antibody-based analysis were used to detect pathogens. Additionally, diverse chromatographic methods were employed to detect toxins based on their chemical and structural properties. However, conventional techniques have several disadvantages concerning analysis time, sensitivity, and expense. With the advances in biotechnology, new approaches to detect pathogens and toxins have been reported to compensate for the disadvantages of conventional analysis from different research fields, including electrochemistry, nanotechnology, and molecular biology. Among them, we focused on the recent studies of transcription factor (TF)-based biosensors to detect microorganisms and discuss their perspectives and applications. Additionally, the other biosensors for detecting microorganisms reported in recent studies were also introduced in this review.

## 1. Introduction

Microorganisms are an integral part of our daily lives and play unique roles inside and outside living organisms and environmental systems. The beneficial effects of microorganisms on human health, the bioremediation of contaminated environments, and the agricultural industry are well-known [1,2]. In addition, soil microbes are an essential part of the forest ecosystem, playing vital roles in sustainable aquaculture [3,4]. Microorganisms also positively impact human health through nutrient cycles and microbiomes [5,6]. On the other hand, some microbes are considered life-threatening because of their ability to cause diseases and produce harmful toxins. Microorganism-contaminated diets and beverages have severe consequences on human health. *Escherichia coli, Salmonella enterica, Campylobacter jejuni*, *Staphylococcus aureus*, *Listeria monocytogenes* and *Bacillus cereus* are considered major foodborne pathogens. Additionally, the toxins produced by microorganisms are also regarded as threats to human health and safety. In this regard, it is pivotal to equip society with methods to detect pathogens and microbial toxins to ensure human health and safety.

Microorganisms originating from diverse sources can be detected by various methods targeting microbial cells, metabolites, toxins, DNA, and biomarkers. The morphological properties of microbial cells can be discerned under light microscopy. Though it is a method to detect microorganisms directly, it takes a relatively long time for incubation. Biomarkers such as surface proteins could be targets for microorganism detection. Together with an antibody specific to the biomarker, microorganisms can be detected by an enzyme-linked immunosorbent assay (ELISA) [7]. The method to detect *Vibrio parahaemolyticus* in seafood was developed using a monoclonal antibody-based ELISA [8]. With advances in nanotechnology, various nanomaterials such as AuNP have replaced antibodies as microbial cell-sensing elements in ELISA-based assays [9,10]. Nucleic acids can also serve as targets for microbial detection. Using polymerase chain reaction (PCR) to identify target genes present was one of the conventional methods to detect microorganisms. In contrast, toxins secreted by microorganisms vary in their physicochemical properties, including sizes, structures, and hydrophobicity. Thus, the elements such as antibodies, aptamers, nanoparticles, and enzymes, which can recognize toxins, were used for their detection [11,12,13]. Most of all, instrument-based assays such as mass spectrometry (MS) and high-performance liquid chromatography (HPLC) are typically used because of their high sensitivity and selectivity [14,15]. However, these assays require expensive instruments, time-consuming processes, and well-trained workers. Therefore, various biosensors based on different technologies are being actively developed to detect microorganisms and their toxins with rapidity and simplicity. 

With increasing demand, biosensors based on diverse technologies such as electrochemical, biological, chemical, and nanomaterial techniques have been reported [16,17,18,19]. Although there are various biosensors, the types are determined by their transduction of the output responses. A basic biosensor consists of target sensing and signal reporting elements. The binding or affinity of sensing elements to target analytes induces changes in reporter elements, converting them into measurable physiological outputs via diverse signal transduction methods. For example, it has been reported that biosensors employ metal nanoparticles and other nanomaterials as sensing elements for detecting pathogens and toxins [18,20]. Although the biosensors in both studies were based on nanomaterials, one used fluorescence signals, and the other used an ELISA-based assay for signal transduction. Recently, a nanosensor based on a cell-membrane-modified field-effect transistor (FET) has been used to detect toxins and pathogens [21]. Briefly, the biomembrane was used as a sensing element for toxins; the interaction between the toxins and the biomembrane transduced changes in local charge distribution at the FET surface, enabling the quantification of pathogens and toxins. Moreover, plasmonic techniques, including surface plasmon resonance (SPR) and surface-enhanced Raman spectroscopy (SERS), have been used to detect pathogens and toxins [22,23]. These instruments also employ sensing elements such as antibodies and nanomaterials on the surface; they detect pathogens and toxins by measuring changes in physicochemical properties upon target binding. Although the biosensors described above provide highly sensitive and accurate measurements, they fail to be cost-effective due to the need for expensive equipment and materials. In this regard, bacterial cell-based biosensors have been touted to overcome these disadvantages.

Bacterial cell-based biosensors (or whole-cell bioreporters) have been intensely investigated for their favorable aspects, such as simplicity, low cost, and convenience compared to instrument-based analysis methods [24,25,26]. The low sensitivity and selectivity of bacterial cell-based biosensors could be enhanced by the genetic engineering of sensing elements and host strains [27,28,29]. Most bacterial cell-based biosensors employ genetic systems responding to external stimuli, including chemicals, heavy metals, and other toxicants, to detect targets [30,31]. Transcription factors (TFs) are regulatory proteins in genetic systems, controlling the transcription levels of a series of genes in the presence of target stimuli. If the promoter regions controlled by TFs are fused to reporter genes, the expression of reporter genes can act as a readout of targets. Consequently, one can quantify targets by measuring the expression level of reporter genes. In this case, the TFs (or regulatory proteins) interacting with the targets act as sensing elements, and the reporter genes are signal transducers. 

As described above, appropriate genetic systems are critical for generating bacterial cell-based biosensors. Bacterial cell-based biosensors for sensing and monitoring environmental toxicants have been extensively reviewed [27,31,32,33]. Primarily, heavy-metal-specific biosensors have been a subject of active investigation due to the identification of genetic systems induced by heavy metals. Moreover, easy transportation of heavy metals into the cells has accelerated these investigations. In addition to heavy metals, small chemical and antibiotic-sensing biosensors have been developed along with the identification of genetic systems [34,35]. To this end, cell-based biosensors for microbial toxins and pathogens can be obtained if appropriate genetic systems are available. However, the fact that direct detection of pathogens is impossible for bacterial cell-based biosensors due to restricted transportation into the cells should also be considered. Thus, the detection of pathogens can be achieved by sensing the strain-specific metabolites and not by sensing pathogens themselves. Apart from the direct detection of pathogens, the metabolites and toxins unable to enter the cells could not be detected by bacterial cell-based biosensors. In fact, the mechanism of target recognition by sensing elements has been a disadvantageous aspect of bacterial cell-based biosensors. However, this limitation has been partially addressed by advances in cell-free gene expression systems [36,37]. Unlike bacterial cell-based biosensor systems, sensing the targets and signal transduction occur outside of cells in cell-free systems. The cell-free systems were expensive and sophisticated to process but could circumvent issues related to target entry in the cells.

In this review, we focus on transcription factor (TF)-based biosensors, including the bacterial cell-based biosensor and the cell-free biosensor systems owing to significant inroads made in their investigation and development. To facilitate an understanding of the designs and principles of transcription-factor-based biosensors systems, we explain the sensing and reporting elements as well as genetic engineering methods to modulate the performances of biosensors. Then, we review recent studies on detecting pathogens by TF-based biosensor systems. We also introduce technologies other than TF-based biosensors recently reported to detect pathogens and microbial toxins. Lastly, we highlight the applications and prospects of TF-based biosensors for detecting pathogens and toxins.

## 2. Transcription Factor-Based Biosensors

The TF-based biosensors employ TFs as sensing elements. The genes encoding enzymes and fluorescent proteins regulated by TFs are used as reporting domains to transduce output signals. The target analytes of biosensors are determined by the selectivity of TFs playing roles as sensing elements [30,38]. With the advances in biotechnology, the information about the pairs of TFs and their targets is steadily accumulating. Researchers have employed these to develop diverse biosensors for environmental detection, food safety inspection, disease diagnosis, and other fields [39,40,41]. The targets of TF-based biosensors are diverse because cells possess the corresponding mechanisms that can detect external stimuli, including environmental toxicants such as heavy metals and chemicals, microbial toxins, pathogens, and others. Nonetheless, the specific analytes without proper genetic systems could not be targets of TF-based biosensors, and it could be a most disadvantageous aspect of TF-based biosensors over instrument-based analysis. For example, the contamination of Mn(II) could be monitored by ICP-MS but not by TF-based biosensors. Table 1 summarizes the TF-based biosensors reported with the sensing elements and their targets. The TF-based biosensors can be divided into cell-based biosensors and cell-free biosensors. In recent decades, the design of TF-based biosensors is mainly based on living cells such as mammalian, plant, and microbial cells. Since cells possess all components for cellular metabolism, cell-based biosensors are simple, rapid, and convenient. However, one major weakness is the need for the analytes to cross over the cell membrane for detection. On the other hand, this weakness is solved by using cell-free system-based biosensors. Both biosensors have pros and cons but share a common working mechanism to detect targets. In the following sections, we address the working mechanisms of TF-based biosensors and introduce the recent achievements of TF-based biosensors for detecting pathogens.

### 2.1. Principles of TF-Based Biosensors

The most critical parts of TF-based biosensors are the genetic systems induced by external stimuli and the recognition mechanisms of sensing elements and signal reporting elements. One such system helps to design and construct the plasmids carrying the recombinant genes consisting of the promoter and reporter genes, and TFs control the expression of reporter genes. The presence of analytes activating or suppressing the TFs is indicated by reporter gene expression. The signals from reporter genes are transduced to various types of outputs depending on the properties of reporter genes. We describe the common working mechanisms and the differences between cell-based and cell-free biosensor systems below.

### 2.2. Bacterial Cell-Based Biosensors

**Figure 1:**
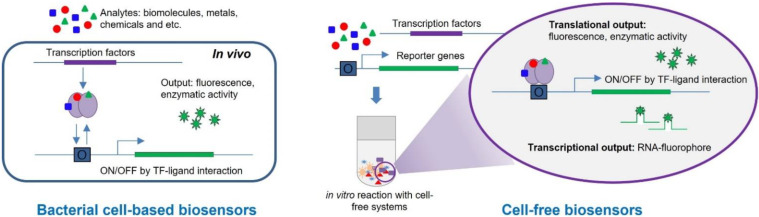
Illustration for the working mechanisms of TF-based biosensors. The cell-based biosensors use the cellular machinery to sense and to report signals as translational outputs (**left**). The biosensors based on cell-free systems use prepared cellular components to sense in vitro and employ both translational and transcriptional outputs as reporting signals (**right**).

Bacterial cell-based biosensors, also called whole-cell bioreporters, have been developed intensively during the last few decades and are regarded as an alternative tool for monitoring hazardous materials in our life [25,61,62]. With recent advances in biological technology, the applications of engineered whole-cell biosensors have expanded to environmental and medical fields for monitoring and detecting toxicants [63,64]. Whole-cell biosensors are microbial cells possessing target sensing domains and reporter domains corresponding to receptors and transducers, respectively (Figure 1). Typically, the regulatory parts of genetic systems responsive to external stimuli are employed as sensing domains, and the genes encoding enzymes and fluorescent proteins are reporter domains. Since the expression levels of reporter domains are regulated by the interaction between targets and sensing domains, the levels of reporter genes correspond to the concentration of the targets. 

Most of the whole-cell biosensors reported employ genetic systems responsive to external stimuli, including heavy metals, chemicals, and other environmental changes. The genetic systems respond to external stimuli by initiating the transcription of a series of genes. The biological mechanism is regulated by certain regulatory proteins recognizing external stimuli (see Table 1). For example, ArsR is a regulatory protein in an arsenic-responsive operon in *E. coli*, and it controls the transcription of *ars*-operon genes in the presence of arsenic [65]. The promoter region of the *ars*-operon is fused with the reporter gene, and the reporter gene expression is controlled by ArsR. Thus, the biosensors employing *ars*-operon have been reported as arsenic-specific biosensors, with the target selectivity dependent on the affinity of ArsR [45,66]. As described here, it is clear that the target sensing relies on regulatory proteins. In this aspect, it was inferred that the target sensing ability of the biosensor could be modulated by changing the regulatory protein. It had been reported that the modulation of target sensitivity and selectivity of bacterial cell-based biosensors was achieved by genetic engineering on regulatory proteins and host cells [28,67]. The antimony sensing biosensor was obtained by genetic engineering of ArsR, and the copper sensitivity was enhanced by deleting *copA* encoding a Cu(II) exporting protein in *E. coli* cells. The performance of bacterial cell-based biosensors was also improved by gene circuit engineering. Jia et al. have reported that the rearrangement of the genetic circuit of the lead resistance operon *pbr* improved the lead sensitivity by about ten times [68]. Moreover, it has also been reported that the detection range and sensitivity to analytes are modulated by the feedback regulation of genetic circuits [69].

Although whole-cell biosensors are being actively developed, the applications for detecting pathogens and toxins are relatively few. The direct interaction between targets and sensing domains triggers the transcriptional initiation of reporter genes. Due to the nature of whole-cell biosensors, the responses of reporter genes only occurred when the target was present inside cells. Therefore, the target of whole-cell biosensors was limited to small molecules that could cross the bacterial cell membrane. Hence, the biological/chemical properties of targets were crucial criteria taken into consideration for constructing and designing the biosensors. These limitations could explain the lack of an active application of whole-cell biosensors to detect microorganisms and toxins compared to other environmental toxicants. Nonetheless, the favorable aspects of whole-cell biosensors, such as low cost, portability, simplicity, and environmental capability, serve to make them attractive as alternative analytic tools over chemical or physical analytical techniques.

### 2.3. Biosensors Based on Cell-Free Systems

The principle of cell-free biosensors is the same as whole-cell biosensors, except for the sensing processes carried out in test tubes. Both types of biosensors share similar genetic systems consisting of sensing and reporter domains, as listed in Table 1. The target recognizing sensing domains are TFs as regulatory proteins, and these proteins regulate the expression of reporter genes in the presence of targets. The most beneficial aspects of a cell-free system over whole-cell biosensors are the absence of limitations for target permeability in cells and diversifying the sensing and signal output elements, including riboswitch, aptamer, and RNA transcript, in addition to the translated proteins [40,58,70]. On the other hand, the high cost of cell-free systems was disadvantageous. Nonetheless, it was widely applied to develop target-sensing biosensors with advances in engineering on diverse molecular structures for sensing elements.

The cell-free system used cell lysates containing the factors necessary to initiate transcription and translation [37,71]. By mixing cell extracts, sensing domains, and reporter domains, target detection was determined by the translation or transcription of reporter genes (Figure 1). The TFs used for cell-based biosensors are employed as sensing elements for TF-based cell-free biosensors to detect specific targets. Similar to cell-based biosensors, the specificity and selectivity rely on the TFs. Therefore, the strategies suggested for enhancing the performance of cell-based biosensors could be applied to cell-free biosensors. When analytes are present, the responses of TFs are induced; they initiate the transcription of reporter genes upon ligand interaction. The output of the signal could be fluorescence, enzymatic activity, or metabolites produced by reporter genes. Moreover, it has also been reported that the transcripts acted as signal reporting outputs by cooperating with fluorescence chemicals.

Since TFs corresponding to heavy metals have been identified, the cell-free biosensors for detecting heavy metals have been reported by many research groups [72,73,74]. Recently, Beabout et al. reported heavy metal biosensors based on cell-free expression systems. They employed metal-responsive TFs such as ArsR, CadC, and MerR as sensing elements for cell-free biosensors to detect As, Cd, and Hg, respectively. The sensing performances of biosensors, such as selectivity and specificity, were enhanced by tuning relative concentrations of sensing and reporter elements [72]. To the same extent, the ability of the cell-free biosensors to detect environmental contaminants such as antibiotics and small chemicals was also actively investigated. Recently, the RNA output sensor activated by the ligand induction (ROSALIND) platform has been reported [58]. The researchers demonstrated the effectiveness of a cell-free biosensor for tetracycline detection in contaminated water by employing TetR, a transcription factor regulating *tet*-responsive operon, as a sensing element. However, the output signal was generated by a fluorescence-activating aptamer rather than by the translation of reporter genes. Additionally, they showed the application of ROSALIND systems to create various cell-free biosensors to detect macrolides, small molecules, and metals by replacing genetic systems and TFs. These studies emphasized that biosensors based on cell-free systems possess a powerful potential to generate new target sensors. In addition to these, various cell-free biosensors have been actively investigated. Although the basic principles of both cell-based and cell-free biosensors are similar, the pros and cons are also distinguishable. Nonetheless, the versatile nature of available sensing and reporting elements for cell-free biosensors would offer great advantages over cell-based biosensors.

## 3. TF-Based Biosensors for Detecting Pathogens

As discussed above, TF-based biosensors have been actively investigated, developed, and implemented in real-world applications. Initially, the major targets of biosensors had been environmental toxicants that harm human health. Recently, the focus has shifted to a wide variety of analytes and advancing sensing element and reporting element design. Although the conventional methods based on analytical instruments are still major tools for detection and monitoring, the need for biosensors has increased because of advantages such as simplicity, cost-effectiveness, and rapidity. However, real-world, practical applications were considered the most imminent obstacles to TF-based biosensors. Though many research groups have put their efforts into developing and applying biosensors, they were restricted at the laboratory scale. In this regard, we discuss the application of TF-based biosensors, especially on pathogens, below.

### 3.1. Bacterial Cell-Based Biosensors

The adverse effects of pathogenic microorganisms have been considered a threat to human health. In this regard, it is crucial to detect and monitor pathogens. Diverse DNA analysis and antibody- and nanomaterial-based techniques have been developed. However, the direct detection of pathogenic cells was hampered in TF-based biosensors due to the nature of their sensing. So far, the sensing mechanisms of TF-based biosensors are based on direct interactions of targets with the sensing domain. Next, the interactions trigger the transcription of reporting elements. Thus, it was hard for whole-celled pathogens to turn on the TF-based biosensors, and there was no recent report despite their many advantages. Since the direct detection was unfeasible, the TF-based biosensors succeeded in detecting the pathogens by indirectly sensing metabolites. Briefly, if the pathogen-specific metabolites, including the quorum sensing molecules and the genetic system responding to them, were available, the TF-biosensors for pathogen detection could be constructed. In Table 2, the quorum-sensing molecules, bacterial species, the responding genetic systems, and the corresponding TF-based biosensors are summarized.

Recently, many TF-biosensors have been developed for detecting pathogenic microorganisms by targeting quorum sensing (QS) molecules [76,87,88]. Quorum sensing is the ability to detect and respond to cell population density by gene regulation [89,90]. Bacterial cells communicate using secreted chemical molecules to coordinate the behavior of the population. For example, the LuxI/LuxR bioluminescence system in *Vibrio fischeri* or the LasI/LasR virulence system in *Pseudomonas aeruginosa* are well-characterized quorum sensing circuits. The former uses *N*-(3-oxohexanoyl)-homoserine lactone (HSL), while the latter uses *N*-(3-oxododecanoyl)-homoserine lactone as quorum-sensing molecules, respectively [90,91]. Based on quorum sensing systems from different microorganisms, several biosensors detecting *N*-Acyl homoserine lactones (AHLs) have been developed [78,92,93]. However, these biosensors focused on detecting molecules of pathogenic origin to diagnose and manage various bacteria-related disorders. The quorum-sensing molecules were regarded as potential biomarkers of diseases. To the same extent, the detection of microbial cells, including pathogens, would be achieved by identifying species-specific quorum-sensing molecules. Recently, Wu et al. reported pathogen-sensing whole-cell biosensors [76]. The whole-cell biosensor employing the QscR quorum-sensing system could detect *Pseudomonas aeruginosa* and *Burkholderia pesedomallei* in contaminated water. Instead of using LasR, the QscR, a homolog of LasR, was employed as a sensing domain [94]. The QscR was used as a sensing domain to interact with AHLs; *egfp* transcription was induced in the presence of AHLs. Thus, the expression level of eGFP indicated the levels of AHLs, thereby detecting target bacterial pathogens. Additionally, they demonstrated pathogen detection using QscR as a sensing domain, and the lycopene biosynthesis pathway was employed as a reporter domain.

Although the biosensors for detecting pathogenic microorganisms have not been extensively investigated, they were shown to have huge potential to detect various pathogens and microbial cells when the strain-specific quorum-sensing molecules and corresponding genetic systems are identified. So far, whole-cell biosensors have focused on environmental toxicants. Still, they would be promising tools for pathogen detection due to their many advantages over other analytical tools.

### 3.2. Cell-Free Biosensors

Similar to whole-cell biosensors, cell-free biosensors were also applied to detect pathogenic microorganisms by sensing metabolic molecules, including quorum sensing molecules, rather than by directly sensing pathogenic cells. In this regard, the cell-free biosensors for pathogens were constructed by implanting the working systems, including sensing and reporting elements employed in the cell-based biosensors in cell-free systems. As listed in Table 2, the quorum-sensing molecules such as *N*-Acyl homoserine lactones (HSLs) are recognized by sensing elements including LuxR, ScrbR, PhzR, and LasR. Thus, the cell-free systems with a pair of genes acting as sensing and reporting elements were developed as cell-free biosensors for detecting QS molecules [60,82,87,95]. For example, Wen et al. constructed a cell-free biosensor for detecting QS molecules in *Pseudomonas aeruginosa* from human sputum samples [96]. Since *P. aeruginosa* produces N-3-oxo-dodecanoyl-homoserine lactone (3OC12-HSL) recognized by LasR, the expression of *gfp* under P_lasRV_ represented the presence of QS molecules, indicating *P. aeruginosa* infection in human samples. Based on similar principles, many reports on cell-free biosensors for detecting QS molecules originating from microorganisms exist. However, whether the QS molecule sensing would indicate pathogen detection should be considered. In fact, the bacterial species share a common structural moiety of QS molecules, but the major signaling molecules differ from species to species [97,98]. Therefore, if a species-specific QS molecule were identified, the detection of QS molecules would serve as a proxy for pathogen detection.

As discussed here, cell-free biosensors have been investigated and actively applied to detect and monitor pathogens. The working mechanism rendered the detection indirect; cell-free biosensors could be used for pathogen detection by sensing the metabolites, including QS molecules and nucleic acids. Although the application of cell-free biosensors was restricted to the microorganisms whose biomarkers were well known, it shows immense potential for unlimited targets if strain-specific biomarkers are identified. In this regard, the future of cell-free biosensors could be significantly developed and expanded not only to medical diagnosis and clinical tests but also to various industrial fields.

## 4. Other Types of Biosensors for Detecting Pathogens

In addition to TF-based biosensors, many different types of biosensors have been reported to detect pathogens and toxins from varied research areas. The biosensors rapidly detect microbial cells, toxins, and DNA by converting the affinity binding of a target into a measurable physical output via various signal transduction methods [99,100]. The biosensors classified as electrochemical biosensors employ various electrochemical processes such as potentiometry, amperometry, voltammetry, and conductometry, for detecting analytes [101,102]. Although the biosensors based on different techniques were categorized into different types, they all share common components, such as the sensing elements and signal reporting transducers. The sensing elements could be immobilized DNA, antibody, aptamers, and nanostructures according to target analytes. The electrochemical signal transducers adopted were determined by the interaction properties of sensing elements and analytes. Here, we introduce several new and recently reported techniques other than TF-based biosensors for detecting pathogens.

Concerning sensing elements, the nanomaterial-based biosensors were actively investigated to elucidate their pathogen-detecting ability. Nanomaterial-based pathogen detection by biosensors follows similar principles to those of other biosensors. The detection of specific bacterial strains relies on the molecular interactions between biological molecules on bacterial cell walls and sensing elements such as an antibody, aptamer, or biological chemicals. Sensing elements conjugated with nanomaterials recognize target analytes, and the interactions induce electrochemical, fluorometric, and colorimetric changes as output signals. Thus, it is noticed that the specificity of biosensors is determined by the target recognition of sensing molecules and the sensitivity by signal transduction processes. There are several intensive reviews on nanomaterial-based biosensors for detecting microbial toxins and pathogens [103,104,105], and they can be subdivided based on signal-transducing techniques.

Nucleic acids have been used as biomarkers to identify microorganisms by analyzing DNA sequences. Unlike conventional analysis, nucleic acids were employed as sensing elements of biosensors to detect microorganisms. The functional nucleic acid (FNA)-based biosensors were recently reported to detect pathogens [106,107]. The FNAs such as DNAzymes and aptamers were employed as sensing elements for pathogen detection. Liu et al. reported using RNA-cleaving fluorescent DNAzymes (RFDs) for pathogen detection [108]. If a DNAzyme responding to a specific pathogen or its metabolites was engineered, the fluorescence of RFDs could be induced by cleaving the quencher near the fluorophore. Consequently, the detection of pathogens was indicated by measuring the fluorescence signals. Although the aptamers were included as sensing elements in nanomaterial-based biosensors, the aptamers could also act as biosensors for pathogen detection by integrating other systems [109,110]. Pathogen-sensing aptamer-based biosensors were constructed with rolling circle amplification (RCA). Briefly, a fluorescence-labeled aptamer initiates RCA in the presence of a target pathogen, generating a DNAzyme capable of producing a colorimetric readout. So far, many aptamers specific to pathogens have been reported as pathogen sensing elements. With advances in biotechnology, nucleic acids recognizing specific pathogens can be implemented in new biosensors as sensing elements, integrating techniques in other research fields.

**Figure 2:**
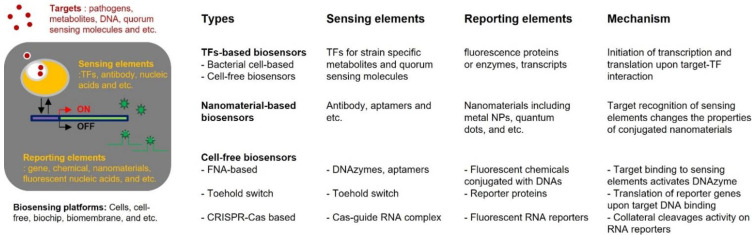
The components and sensing mechanisms of different biosensors for pathogen detections. The common mechanisms of biosensors for detecting pathogens were illustrated, and the components and working mechanisms of different types of biosensors were listed.

In addition to TF-based cell-free sensors, other types of cell-free biosensors have been reported that have new techniques for sensing and reporting elements. They are classified as nucleic-acid-based cell-free biosensors. The toehold switch-based sensors were developed as cell-free biosensors to detect environmental toxicants and pathogens, including diverse viruses [111,112]. Here, nucleic acids were targets for cell-free biosensors, and RNA-based switches acted as sensing elements for target nucleic acids. The transcripts were inactivated by forming RBS sequestered hairpin structures, and then the reporter genes were translated upon target RNA assembly [40]. Moreover, the toehold switch systems have provided a superior detection limit [113]. The toehold switch-based cell-free biosensors have been applied to the medical field to provide point-of-care monitoring [114,115,116]. It has been reported that Ebola, Zika, and SARS-CoV-2 viruses were detected in patients’ samples by the toehold switch-based biosensors. They could be applied to detect diverse microorganisms if the strain-specific nucleic acid sequences were determined. By integrating target sequences in sensing elements, the biosensors could detect the presence of microorganisms in samples by measuring the expression of reporter genes. In addition, novel CRISPR-associated enzymes with different target specificities and activities have contributed to the development of methods such as the DNA Endonuclease-Targeted CRISPR Trans Reporter (DETECTR) and the Specific High-Sensitivity Enzymatic Reporter UnLOCKing (SHERLOCK) [117,118,119]. The detection of diverse pathogens such as African swine fever, influenza A and B, Zika, and Dengue virus has been reported based on the DETECTR and SHERLOCK systems [118,120,121,122]. Moreover, they have been intensively investigated because of their rapid and accurate detection with superior sensitivity. Although the details about these new methods for the detection of pathogens are not discussed in this review, they also showed the similarity in target sensing mechanisms to other biosensors. We illustrated the common mechanisms of pathogen-sensing biosensors and listed the sensing and reporting elements for each type of biosensors mentioned in this review in Figure 2.

## 5. Conclusions

Here, we introduce biosensor systems focused on TF-based biosensors for detecting pathogens. TFs are the regulatory proteins for certain genetic systems, and they turn the genetic systems on or off in the presence of targets. Thus, the TFs were employed as sensing elements recognizing target materials, and the transcription of reporter genes controlled by TFs indicated the presence of target materials. The TF-based sensing systems worked as biosensors in cells, and cell-free expression systems were categorized as cell-based and cell-free biosensors, respectively. Both sensing systems possess pros and cons, but they share the same genetic systems for designing biosensors. At this moment, it is hard to designate a better biosensor system, but one could be selected based on the physicochemical properties of targets. Most of all, target-recognizing sensing elements are critical for TF-based biosensors. If the appropriate TFs corresponding to targets were available, it would be possible to construct new target-specific biosensors. In this regard, the TF-based biosensor systems may not be the best method for detecting pathogens because of the necessity of TF-target interactions. However, we foresee the expansion of TF-based biosensors for pathogens along with the accumulation of data for strain-specific biomarkers with the advances in biotechnology. In addition, we also allude to recent techniques for sensing pathogens such as nanomaterial-based, nucleic acid-based, and cell-free biosensor systems, including toehold switch-based biosensors, such as the DETECTR and SHERLOCK systems. These biosensors employ nanomaterials, nucleic acid (DNA/RNA), and aptamers as sensing elements for detecting targets.

Although many different types of biosensors have been developed for pathogens, their applications to real fields are still limited because of the gap between laboratories and industries. It was proven that the performances of the biosensors for pathogen detection, including TF-based, nanomaterial-based, and nucleic acid-based biosensors, were comparable to the conventional methods and even better in terms of the specificity and sensitivity. However, several challenges such as the expenses, the production of materials, and the requirement of specialized equipment would be obstacles to commercializing biosensors. In addition, the verification or validation of new methods by safety/health authorities is crucial. Nonetheless, we believe the biosensors would be used more widely as alternative tools for detecting pathogens due to their advantageous aspects. The challenges will be overcome in the near future by the efforts of researchers, and the on-site application will be achieved soon with rigorous efforts toward the construction of portable biosensor devices [123,124,125].

Conclusively, we are all aware that the adverse effects of pathogens threaten human health, and it is pivotal to have better methods to detect them. Though conventional methods were capable of monitoring and detecting pathogens with reliable accuracy, it was necessary to develop simple, cheap, rapid, and convenient methods that would help to detect harmful pathogens. In this regard, the biosensor systems possess great potential to be an alternative method for detecting pathogens as well as environmental toxicants and pollutants.

## Figures and Tables

**Table 1:** Transcription factor-based biosensors.

Types	Analytes	Genetic Systems		Output Elements	Refs
		Bacterial Strains	TFs		
Whole-cell biosensors	Cu(II)Ag(I)	*E. coli*	CueR	luxCDABE	[42]
	Pb(II)Hg(II)Zn(II)Cd(II)	*E. coli*	ZntR	luxCDABE/eGFP	[42,43]
	As(III)As(V)	*E. coli*	ArsR	Luciferase/β-galactosidase/ GFP	[44,45]
	Benzoate	*P. putida*	BenR	GFP	[46]
	Malonyl-CoA	*B. subtilis*	FapR	eGFP	[47]
	BTEX (benzene, toluene, ethylbenzene, xylene)	*R. Pickettii*	TbuT	GFP	[48]
	Sodium Dodecyl Sulfate(SDS)	*P. aeruginosa*	SdsB1	GFP	[49]
	Lactate	*E. coli*	LldR	GFP	[50]
	Homogenitisic Acid	*P. aeruginosa*	HmgR	GFP	[51]
	2,4-diacetylphloroglucinol(DAPG)	*P. fluorescens*	PglF	LacZ/ luxCDABE	[52]
	Salicylate	*P. putida*	NahR	luciferase	[53]
	Trans-cinnamic Acid	*E. coli*	HcaR	eYFP	[54]
	Caprolactam	*A. faecalis*	NitR	sfGFP	[55]
	Salicylic acid	*E. coli*	MarR	eGFP	[56]
Cell-free biosensors	Hg(II)	*S. flexneri*	MerR	sfGFP	[57]
	γ-hydroxybutyrate	*A. tumefaciens*	BlcR	sfGFP	[57]
	Tetracycline	*E. coli*	TetR	ROSALIND:Transcript-fluorophore complex	[58]
	Oxytetracycline	*S. rimosus*	OtrR		
	Erythromycin	*E. coli*	MphR		
	3-hydroxy benzoic acid	C. testosteroni	MobR		
	Zn(II)	S. elongatus	SmtB		
	Cu(I), Cu(II)	B. subtilis	CsoR		
	Cd(II)	S. aureus	CadC		
	Pb(II)	S. aureus	CadC		
	As(III)	*E. coli*	ArsR	ArsR-GFP released from immobilized DNA upon As(III)	[59]
	Benzoic acidHg(II)As(III)	*E. coli*	BenRMerRArsR	eGFP	[60]

**Table 2:** TF-based biosensors detecting quorum sensing molecules.

QS Molecules	Bacterial Species	Genetic Systems		OutputElements	Expression System	Refs
		Promoters	TFs			
Homoserine lactones and N-acyl homoserine lactones(HSLs and AHLs)	*P. aeruginosa*	*rsaL*	LasR	luxCDABE	*P. aeruginosa*	[75]
	*P. aeruginosa,*	*PA1897*	QscR	luxCDABE	*E. coli*	[76]
	*V. fischeri*	*luxI/R*	LuxR	luxCDABE	*E. coli*	[77]
	*P. aeruginosa*	*rhlI*	RhlR	luxCDABE	*E. coli*	
	*P. aeruginosa*	*lasI*	LasR	luxCDABE	*E. coli*	[78]
	*A. tumefaciens*	*traCDG*	TraR	lacZ	*A. tumefaciens*	[79]
	*P. fluorescens*	*phzA*	PhzR	lacZ	*P. fluorescens*	[80]
	*P. syringae*	*ahlI/ahlR*	AhlR	eGFP/mCherry	*E. coli*	[81]
	*S. coelicolor*	*scbR/scbA*	ScbR	GFP	Cell-free	[82]
	*P.aeruginosa*	*lasRV*	LasR	GFP	Cell-free	[83]
Autoinducer peptides	*S. aureus*	*agrA*	AgrA/AgrC	GFP/Lacticin	*E. coli*	[84]
Autoinducer-2	*V. harveyi BB170*	*lux*	LuxR	luxCDABE	*V. harveyi BB170*	[85]
Gelatinase biosynthesis activating pheromone	*E. faecalis*	*gelE* *fsrB*	CylR1CylR2	luxCDABE	*E. faecalis*	[86]
Extracellular death factor	*E. coli*	*mazEF*	MazEF	-	*E. coli*	[83]

## Data Availability

Not applicable.
